# Deciphering the Key Pharmacological Pathways and Targets of Yisui Qinghuang Powder That Acts on Myelodysplastic Syndromes Using a Network Pharmacology-Based Strategy

**DOI:** 10.1155/2020/8877295

**Published:** 2020-12-08

**Authors:** Zijian Han, Luping Song, Kele Qi, Yang Ding, Mingjun Wei, Yongcun Jia

**Affiliations:** ^1^People's Hospital of Ningxia Hui Autonomous Region, Department of Medical Engineering, Yinchuan 750002, China; ^2^The First Affiliated Hospital of Northwest University for Nationalities, Yinchuan 750002, China; ^3^People's Hospital of Ningxiang, Department of Clinical Pharmacy, Changsha 410600, China; ^4^People's Hospital of Ningxia Hui Autonomous Region, Department of Emergency Medicine, Yinchuan 750002, China; ^5^People's Hospital of Ningxia Hui Autonomous Region, Department of Burn and Plastic Surgery, Yinchuan 750002, China; ^6^South China University of Technology, Graduate School, Guangzhou, 510006, China; ^7^People's Hospital of Ningxia Hui Autonomous Region, Department of Clinical Laboratory, Yinchuan 750002, China

## Abstract

**Background:**

Yisui Qinghuang powder (YSQHP) is an effective traditional Chinese medicinal formulation used for the treatment of myelodysplastic syndromes (MDS). However, its pharmacological mechanism of action is unclear.

**Materials and Methods:**

In this study, the active compounds of YSQHP were screened using the traditional Chinese medicine systems pharmacology (TCMSP) and HerDing databases, and the putative target genes of YSQHP were predicted using the STITCH and DrugBank databases. Then, we further screened the correlative biotargets of YSQHP and MDS. Finally, the compound-target-disease (C-T-D) network was conducted using Cytoscape, while GO and KEGG analyses were conducted using R software. Furthermore, DDI-CPI, a web molecular docking analysis tool, was used to verify potential targets and pathways. Finally, binding site analysis was performed to identify core targets using MOE software.

**Results:**

Our results identified 19 active compounds and 273 putative target genes of YSQHP. The findings of the C-T-D network revealed that Rb1, CASP3, BCL2, and MAPK3 showed the most number of interactions, whereas indirubin, tryptanthrin, G-Rg1, G-Rb1, and G-Rh2 showed the most number of potential targets. The GO analysis showed that 17 proteins were related with STPK activity, PUP ligase binding, and kinase regulator activity. The KEGG analysis showed that PI3K/AKT, apoptosis, and the p53 pathways were the main pathways involved. DDI-CPI identified the top 25 proteins related with PI3K/AKT, apoptosis, and the p53 pathways. CASP8, GSK3B, PRKCA, and VEGFR2 were identified as the correlative biotargets of DDI-CPI and PPI, and their binding sites were found to be indirubin, G-Rh2, and G-Rf.

**Conclusion:**

Taken together, our results revealed that YSQHP likely exerts its antitumor effects by binding to CASP8, GSK3B, PRKCA, and VEGFR2 and by regulating the apoptosis, p53, and PI3K/AKT pathways.

## 1. Introduction

Myelodysplastic syndromes (MDS) are a heterogeneous group of hematological disorders characterized by bone marrow (BM) dysplasia, abnormal myeloid cell differentiation, peripheral blood cytopenia, and an increased risk of leukemic transformation [1]. MDS results in poor clinical outcomes, and the duration of patient survival is less than 2 years for the higher risk subtypes [2]. Immunomodulatory drugs (e.g., lenalidomide), hypomethylating agents (e.g., 5-azacytidine and decitabine), and stem cell transplantation are currently approved methods of treatment for MDS [3–5] but are limited due to chemoresistance and adverse effects of the drugs. Therefore, there is an imperative need to develop novel therapeutic strategies against MDS.

Traditional Chinese medicine (TCM) has been used to treat various illnesses for thousands of years and is associated with fewer toxic effects compared with pharmacological drugs [6, 7]. Yisui Qinghuang powder (YSQHP), a TCM formulation used to treat MDS, consists of indigo naturalis (qingdai), red ginseng (hongshen), and realgar (xionghuang) [8, 9], a mineral consisting of arsenic sulfide (As_4_S_4_) and arsenic trioxide (As_2_O_3_). As_4_S_4_ is an insoluble compound, while As_2_O_3_ is a soluble therapeutically active compound [10]. Although the therapeutic effects of YSQHP are encouraging, its pharmacological mechanisms of action are still unknown.

**Figure 1:**
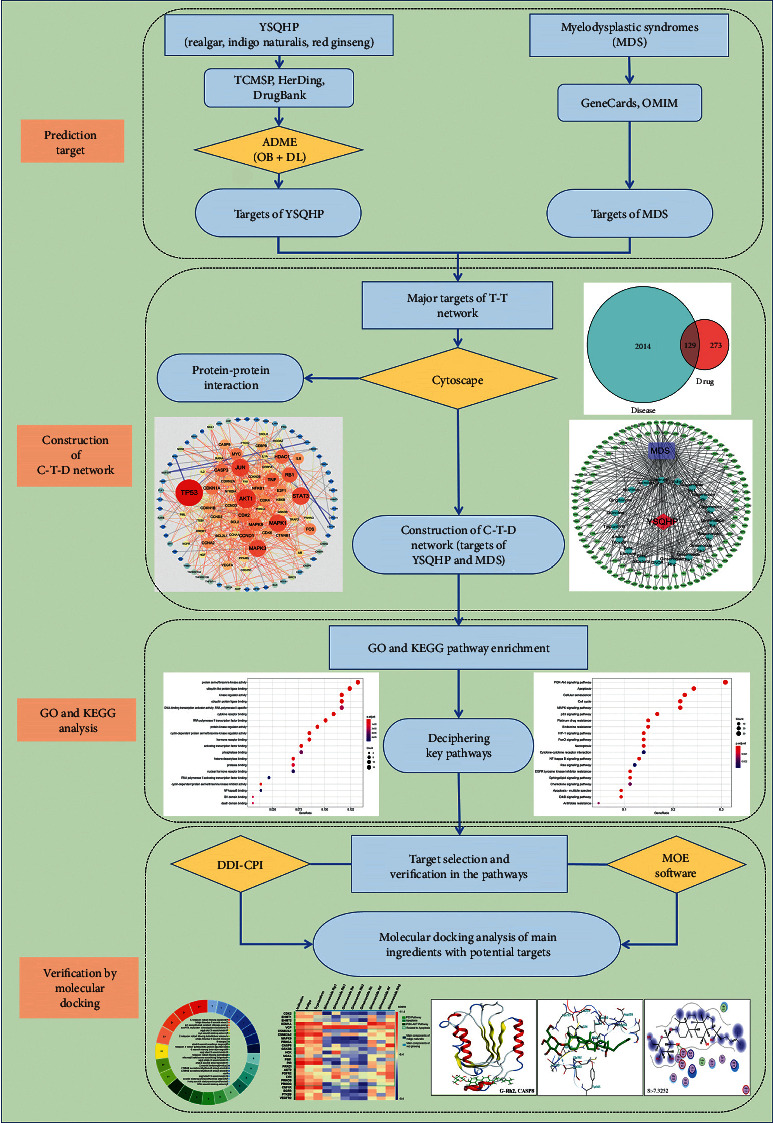
Outline of research strategy.

Network pharmacology integrates systems biology with polypharmacology and is a suitable approach to characterize complex TCM formulations and identify their multiple targets and mechanisms of action [7, 11]. We identified the bioactive compounds of YSQHP using the TCMSP [12] and HerDing databases [13] and established the network of these compounds, their putative targets, and diseases. Gene ontology (GO) and Kyoto Encyclopedia of Genes and Genomes (KEGG) database analyses were then used to elucidate multiple targets and pathways affected by YSQHP in MDS. The signaling pathways elucidated through network pharmacology were validated using DDI-CPI, a web tool based on AutoDock Vina [14] and MOE software (strategies used are shown in Figure 1).

## 2. Methods

### 2.1. Identification of Bioactive Compounds

The constituents of YSQHP were screened for using the traditional Chinese medicine systems pharmacology (TCMSP) (https://tcmspw.com/tcmsp.php) which is a unique systems pharmacology platform of Chinese herbal medicines that captures the relationships between drugs, targets, and diseases. And, HerDing (http://210.107.182.73/TCMIDWebService/herdingDemo.jsp) database is a search engine that recommends herbs to treat diseases using genes and chemicals by integrating existing several databases and a text mining approach.

The pharmacokinetic interactions of the compounds were identified using ADME (absorption, distribution, metabolism, and elimination), and the active compounds of YSQHP were screened for two drug-likeness indices OB (oral bioavailability) value of ≥30% and DL (drug-likeness) value of ≥0.18 for eliminating the ineffective compounds in herbs. Then, the active compounds omitted from the supplementary part were excavated by reading the literature.

### 2.2. Target Prediction of the Active Compounds

The key targets of As_2_O_3_ were identified using the STITCH database (http://stitch.embl.de/) [15] and the DrugBank database (https://www.drugbank.ca/) [16]. Then, information on the active compounds omitted from the supplementary section was extracted by scanning the relevant literature, especially the literature on ginsenosides and the secondary metabolites of ginsenosides. We collected information using the TCMSP and HerDing databases, and the names and chemical structures of the 19 compounds were obtained. All information on the compounds was standardized based on “Canonical SMILES,” which is based on the PubChem database (https://pubchem.ncbi.nlm.nih.gov/).

### 2.3. Screening of the MDS-Related Targets

The potential therapeutic targets of MDS were screened for from the GeneCards (https://www.genecards.org/) and Online Mendelian Inheritance in Man (OMIM) (http://www.omim.org/) databases using the keyword, *myelodysplasia syndrome*. After duplicate data were removed, the proteins obtained from both databases were pooled using R software and converted into UniProt IDs.

### 2.4. Network Construction

The relationship between the active ingredients of YSQHP and the predicted targets and active compounds were visualized by constructing the compound-target-disease (C-T-D) network using Cytoscape (v.3.6.1). The target-target (T-T) network between YSQHP and MDS was constructed using the STRING database (https://string-db.org/) based on a minimum required interaction score of 0.900, and it was visualized using Cytoscape.

### 2.5. GO and KEGG Pathway Analyses

The signaling pathways associated with the therapeutic targets of MDS were then identified using DAVID (Database Visualization and Integrated Discovery software, http://david.abcc.ncifcrf.gov). The genes were functionally annotated through the GO analysis, and significantly enriched terms with a *P* value <0.5 and FDR <0.5 were screened for. The KEGG (http://www.genome.jp/kegg/) database was used to identify enriched pathways, and data were analyzed and displayed using the GO plot package of R software (v.3.6.1). To further elucidate the mechanism underlying the therapeutic effect of YSQHP against MDS, selected targets were annotated for the apoptosis, p53, and PI3K/AKT signaling pathways (KEGG number: map04210, map04115, and map04151) to identify the interacting genes, and the network was drawn using the KEGG parser.

### 2.6. Molecular Docking Simulation

Drug-drug interactions were predicted via chemical-protein interactome or using DDI-CPI (https://cpi.bio-x.cn/ddi/) to simulate molecule docking and predict drugs with similar structure. Eleven effective compounds in YSQHP (arsenic trioxide was not identified in the database; 3 compounds were identified in indigo naturalis; and 8 compounds were identified in red ginseng) were identified, and the structure of these 11 compounds was uploaded to the website for docking. Based on the optimistic model of the data mining results of this website, a docking score that was less than or equal to −8.4 was considered to indicate a potential interaction with the protein. To understand the frequency of the occurrence and the molecular functions of the potential targets, keywords, such as apoptosis, p53, and PI3K/AKT, were used to classify the functions of the proteins. Using the frequency of potential targets in the list, the top 25 proteins were selected and were used in the chemical-protein interactome (CPI) analysis. The molecular docking of indirubin, G-Rh2, G-Rf to GSK3B, CASP8, PRKCA, and VEGFR2 was verified using MOE software (v.2019.0102)..

## 3. Results

**Figure 2:**
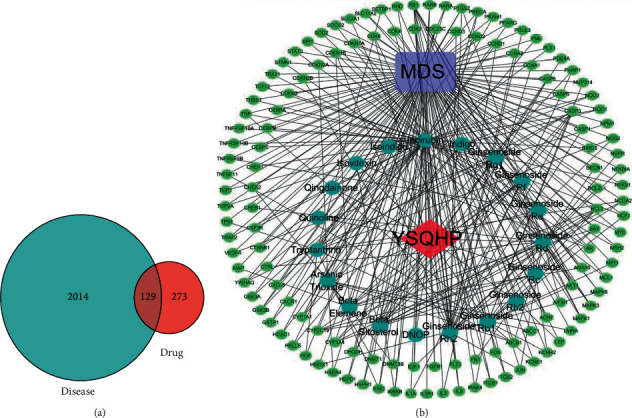
Screening of potential targets and the compound-target-disease network. (a) All MDS targets (2,014) obtained from GeneCards and OMIM, the 273 targets of YSQHP obtained using the TCMSP and HerDing databases, and their intersection (129 genes). Blue: the 2,014 targets related to MDS (disease); red: the 273 targets of YSQHP (drug); dark red: the 129 overlapping targets between MDS and YSQHP. (b) compound-target-disease (C-T-D) network of the 129 overlapping targets of MDS and 19 YSQHP compounds (11 from red ginseng and 8 from indigo naturalis and realgar). Red diamond: YSQHP (drug); blue circles: effective molecules in the drug (19 ingredients); purple square: MDS (disease); green dots: disease-related targets (129 genes); black lines: network relationships.

**Figure 3:**
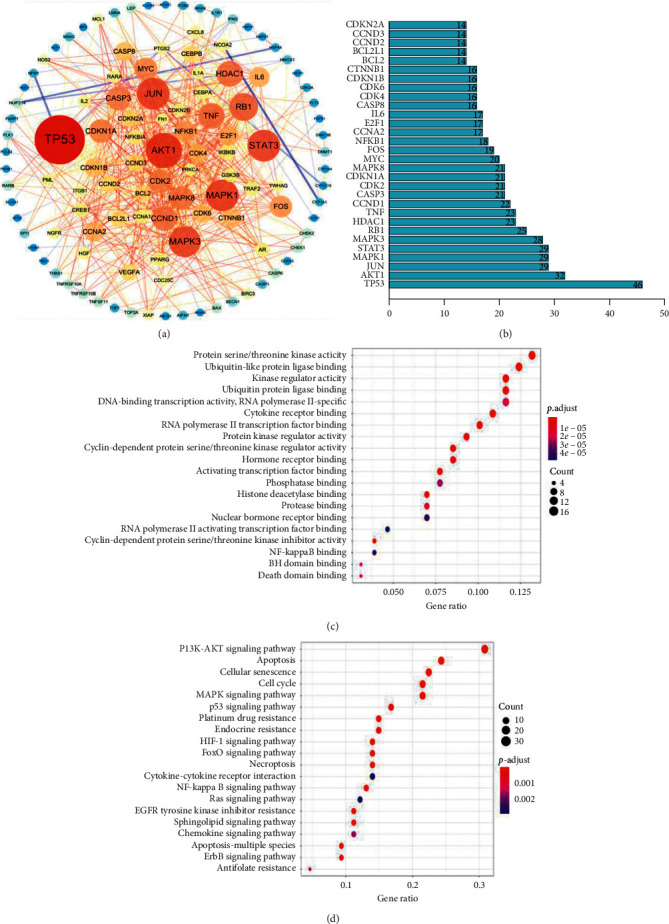
Protein-protein interaction (PPI) and functional annotation of the 129 overlapping proteins. (a) Protein-protein interactions (PPIs) of the 129 targets. Targets in the outer ring have ≤7 edges and are less interactive, while those in the inner ring are highly interactive and have >7 edges. Color and size of the circles: the more the edges, the redder the color and the larger the shape. Color and thickness of the lines: red indicates positive regulation, while blue indicates negative regulation, and the thicker the line, the higher the correlation. (b) Bar chart showing the top 30 PPIs arranged according to the number of edges. (c) GO enrichment analysis showing the top 20 molecular biological functions. The abscissa represents the ratio of genes, and the ordinate lists the protein functions. The bubble size represents the number of proteins, while the color represents the *P* value. (d) The KEGG analysis showing the top 20 pathways. The abscissa indicates the ratio of genes, and the ordinate lists the pathways. The bubble size indicates the number of proteins, and the color indicates the *P* value.

**Figure 4:**
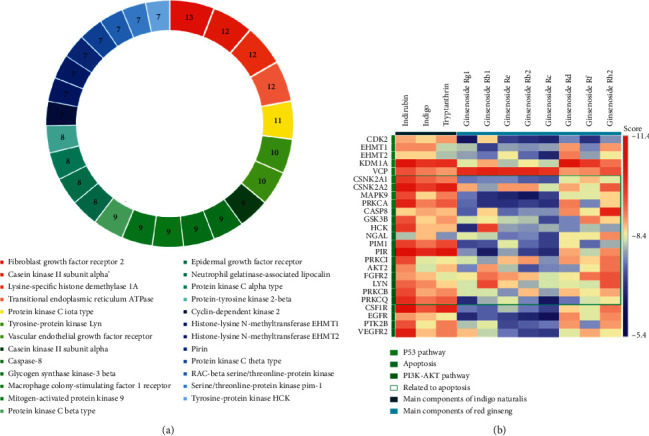
Drug-drug interactions via chemical-protein interactome (DDI-CPI) analysis dock score of the compounds in YSQHP. (a) Top 25 targets of YSQHP in the drug-drug interaction (DDI) obtained via chemical-protein interactome (CPI) analysis. The target proteins are represented using different colors. The number represents the number of active components that interact with the target. (b) Heatmap showing the docking scores of 3 compounds in indigo naturalis and 8 compounds in red ginseng to the top 25 targets. ^*∗*^Apoptosis: proteins directly involved in the apoptosis pathway; related to apoptosis: proteins involved in the apoptosis pathway and other pathways.

**Figure 5:**
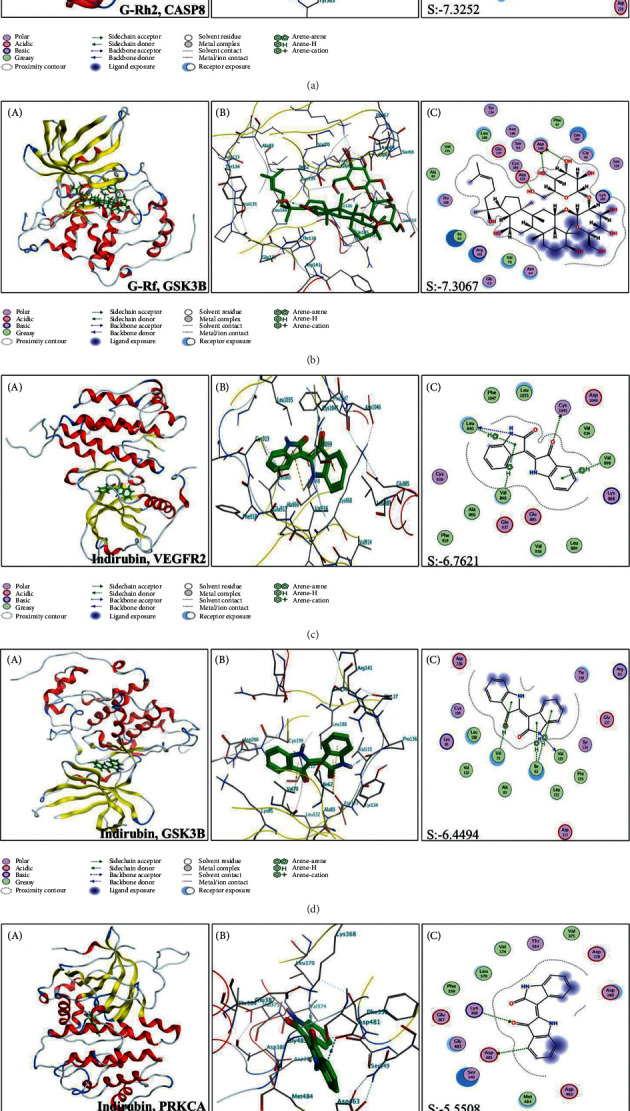
Results of the molecular docking analysis. (Aa-Ea) Overall view of the docking mode of ligands (green) in the receptor binding site (colored ribbons). (Ab-Eb) Site view of potential amino acid residues surrounding ligands (green) in the receptor binding pocket (colored sticks). (Ac-Ec) Two-dimensional interaction map of ligands and receptors. The arrows indicate potential interactions between amino acid residues and ligands. S is the docking score given by MOE software.

  A total of 11 compounds were identified in red ginseng: 9 compounds from TCMSP and 8 compounds from HerDing. Furthermore, 8 compounds of indigo naturalis, 8 compounds from TCMSP, and 3 compounds from HerDing were also identified (see Table 1 for details).
  A total of 273 known targets of YSQHP were extracted from the TCMSP, HerDing, and STITCH databases, and 2,014 MDS-related genes were obtained from the OMIM and GeneCards databases. The Venn plot revealed that 129 MDS-related genes were also targeted by YSQHP (Figure 2(a)). The C-T-D network comprised 150 nodes and 339 edges (Figure 2(b)). Among the MDS-related genes, Rb1, CASP3, BCL2, and MAPK3 showed the most number of interactions, whereas indirubin, tryptanthrin, G-Rg1, G-Rb1, and G-Rh2 showed the most number of potential targets.
  The T-T network comprised 129 nodes and 545 edges, with an average node degree of 8.45 and an average local clustering coefficient of 0.459 (Figure 3(a)). P53, AKT1, STAT3, MAPK1, MAPK3, and JUN were hub genes, while the top 10 protein-protein interaction (PPI) nodes (*P* value <1.0*e* − 16) were TP53, AKT1, JUN, MAPK1, STAT3, MAPK3, RB1, HDAC1, TNF, and CCND1.
  The GO analysis of the 129 overlapping targets showed that protein serine/threonine kinase activity, ubiquitin-like protein ligase binding, kinase regulator activity, ubiquitin protein ligase binding, and DNA-binding transcription activator activity (RNA polymerase II-specific) were the top 5 enriched functions. In addition, 17 proteins were associated with protein serine/threonine kinase activity with a gene ratio of 0.132, while 16 proteins were associated with ubiquitin-like protein ligase binding function (Figure 3(c)). Thus, YSQHP compounds could potentially target these proteins to regulate kinase activity, in particular serine/threonine and ubiquitination.
  The top 5 significantly enriched pathways among the 129 overlapping genes were PI3K/ AKT (33 proteins), apoptosis (26 proteins), cellular senescence (24 proteins), and cell cycle and the MAPK signaling pathways (15 proteins), as shown by the KEGG analysis (Figure 3(d)). The p53 pathway ranked sixth with 18 proteins and is known to play a key role in both the cellular senescence and cell cycle pathways. Since p53 was also a hub protein in the PPI network, we surmised that the p53 pathway is targeted by YSQHP in MDS.
  The top 25 proteins were identified based on the docking score and the target functions (keywords: p53, apoptosis, and AKT) (Figure 4(a)). Furthermore, CDK2, EHMT1, EHMT2, KDM1A, VCP, CSNK2A1, CSNK2A2, MAPK9, and PRKCA were found to be associated with p53. CASP8, GSK3B, HCK, NGAL, PIM1, PIR, and PRKC are likely to be involved in apoptosis. In addition, AKT2, FGFR2, LYN, PRKCB, PRKCQ, CSF1R, EGFR, PTK2B, and VEGFR2 were associated with PI3K/AKT. CSNK2A1, CSNK2A2, MAPK9, and PRKCA were associated with both p53 and apoptosis, whereas AKT2, FGFR2, LYN, PRKCB, and PRKCQ were common to both apoptosis and PI3/AKT. The indigo naturalis compounds had a high molecular docking score (<−8.4) with most of the top 25 targets, while indirbin produced the highest score of −11.4, which corresponds to its interaction with all but NGAL. In red ginseng, G-Rd, G-Rf, and G-Rh2 showed relatively higher scores, compared with G-Rg1, G-Rb1, and G-Re (Figure 4(b)) (see Table 2 for details).
  CASP8, GSK3B, PRKCA, and VEGFR2 were identified through both the DDI-CPI and PPI analyses (Figure 5) and have a high docking score in the DDI-CPI system: CASP8 with G-Rh2: −10.2, GSK3B with G-Rf: −9.1, PRKCA with indirubin: −10.2, and VEGFR2 with indirubin: −10.3, indicating that they are potential core-targets of YSQHP. According to MOE software, indirubin interacts with GSK3B, PRKCA, and VEGFR2, G-Rh2 with CASP8, and G-Rf with GSK3B. The pentasaccharide of G-Rh2 interacts with Lys320 and Asp363 of CASP8 (S: −7.3252 and E refine: −40.5020) and that of G-Rf may bind to Asp133 and Asp200 of GSK3B (S: −7.3067 and E refine: −22.5884). Indirubin showed potential binding with Leu840, Val848, Cys1045, and Val899 of VEGFR2 (S: −6.7621 and E refine: −27.5642) and is likely to bind to GSK3B via arene-H interaction with Val70, Ile62, and Val135 (S: −6.4494 and E refine: −33.7944). PRKCA may also be a potential target of indirubin and interact through Lys368 and Asp481 (S: −5.5508 and E refine: −27.0008).

## 4. Discussion

Network pharmacology is a novel field that integrates pharmacology, biochemistry, genomics, and bioinformatics [7]. It is an effective strategy to visualize and analyze complex interactive data on herbs, compounds, targets, and diseases based on computer modeling analysis and target prediction methods [11] and is especially suitable for characterizing TCM formulations. Furthermore, molecular docking is a powerful method used to predict the binding force between the drug compounds and their putative targets based on the spatial structure of the drug and ligand. PPI-CPI is a web tool based on AutoDock Vina that can analyze putative PK/PD proteins and decipher the potential mechanisms of the DDIs mediated by unexpected drug-protein interactions [14, 17].

MDS is a clonal hematopoietic disorder characterized by bone marrow failure that can develop into acute myeloid leukemia (AML). MDS progression involves multiple signaling pathways [1, 18]. We found that YSQHP potentially targets the apoptosis, cell cycle, p53, PI3K-AKT, and MAPK pathways in MDS. Transcriptional factor p53 is a tumor suppressor that induces downstream senescence or apoptosis-related genes and is frequently deleted or mutated in human malignancies. MDS patients who harbor *TP53* mutations have a significantly higher risk of progressing to AML, compared to those with wild-type alleles [19]. Therefore, the p53 pathway is targeted by multiple anticancer chemotherapeutic drugs [20]. The p53 targets with the highest scores in our analysis were CDK2, EHMT1, EHMT2, KDM1A, and VCP, which usually function as regulators of cell cycle progression [21–23]. EHMT1, EHMT2, and KDM1A, which are histone demethylases, and EHMT1/T2 are targeted in AML to increase hemoglobin F (*α*2*γ*2) levels [21]. VCP is a ubiquitous ATPase that is elevated in several tumors, and its inhibition promotes cancer cell death via autophagy [22]. KDM1A-mediated demethylation of the Lys-370 residue of p53 prevents its interaction with BP1, represses downstream transcriptional activation, and stabilizes the DNA methylase, DNMT1. Furthermore, the RCOR/GFI/KDM1A/HDAC complex epigenetically suppresses genes involved in multilineage blood cell development via histone deacetylase (HDAC) recruitment [18, 24]. The YSQHP compounds, indirubin, indigo, trypenthrin, G-Rd, G-Rf, and G-Rh2 may bind to KDM1A and increase the expression levels of p53, thereby triggering the apoptosis of malignant cells. Since HDAC1 is a target of arsenic trioxide [25], the latter may also affect the RCOR/GFI/KDM1A/HDAC complex.

The molecular docking score of G-Rh2 and CSAP8 was −10.2, indicating that G-Rh2 likely induces CASP8-mediated apoptosis in MDS cells. CASP8 is expressed at low levels in malignant cells, and Xiao-Xi et al. showed that G-Rh2 can promote the apoptosis of *CASP8* knockdown tumor cells via a CASP8-independent pathway. In addition, G-Rh2 inhibits the cisplatin-induced apoptosis of normal renal cells by downregulating the CASP8 expression [26]. Therefore, we surmised that G-Rh2 could remove MDS cells and simultaneously protect normal blood cells by targeting CASP8. The casein kinase 2 (CK2) subunits, CSNK2A1, and CSNK2A2 are overexpressed in multiple tumors and mediate the degradation of the PML oncoprotein [27, 28]. CK2 inhibits cancer cell apoptosis by blocking CASP9 cleavage and CAP8 activation [29]. PRKCA and PRKCB are potentially associated with VEGFA-dependent vascular tube formation [30, 31]. High PRKCA levels in leukemia cells protect the cells from apoptosis via BCL2 activation [32]. Indirubin, indigo, and tryptanthrin may potentially bind to and inhibit PRKCA, PRKCB, PRKCI, LYN, HCK, PIM1, and GSK3B expressions, thereby restoring normal hematopoietic function. Rh2, Rf, Rd, and Rb1 showed weaker binding than those mentioned above and may play an auxiliary therapeutic role. PRKCI is essential for BCR-ABL oncogene-mediated chemoresistance in leukemia cells and prevents drug-induced apoptosis [33, 34]. YSQHP may affect angiogenesis by regulating PRKCA, PRKCB, and PRKCI levels. The Src family of kinases (SFKs), LYN, and HCK have also been associated with the progression of hematopoietic malignancies. LYN is a negative regulator of hematopoiesis that is aberrantly expressed in AML cells as opposed to normal hematopoietic progenitors. Constitutive activation of LYN in erythroid progenitors leads to major compensatory changes in the erythroid compartment [35, 36]. HCK is overexpressed in Waldenstrom macroglobulinemia (WM) and other hematological malignancies and is targeted by Ibrutinib for the induction of apoptosis in chronic lymphocytic leukemia [37, 38]. PIM1 is aberrantly expressed in several cancers, and its pharmacological inhibition in breast cancer, T-ALL cell lines, adult T-cell leukemia, and T-cell lymphoma (ATL) is therapeutically effective [39, 40]. GSK3B inhibition in nonhuman primate-induced pluripotent stem cells (NHP-iPSCs) promotes the generation of myeloid and lymphoid progenitors [41].

Serine/threonine kinase AKT mediates several pathways that regulate metabolism, proliferation, cell survival, growth, and angiogenesis. The AKT2/PI3K pathway is frequently activated in cancer cells and inhibits apoptosis [42]. Given the high levels of activated AKT2 in MDS patients, it is a promising new therapeutic target for MDS and AML [43]. FGFR2 had the highest docking score in our study and was identified as a target of 13 YSQHP compounds. FGFR2, VEGFR2, CSF1R, and EGFR are tyrosine kinases that are constitutively activated in cancer cells to promote tumor progression and angiogenesis. Tyrosine kinase inhibitors (TKIs) are the most commonly used drugs against blood malignancies [44]. CSF1R is expressed in monocytes/macrophages and promotes cell proliferation and differentiation via the PI3K/AKT and MEK/ERK pathways [45]. Monocytes and macrophages are abundant in the marrow of AML patients, and the tumor-associated macrophages (TAMs) are crucial for tumor cell survival and proliferation. Tumor-infiltrating macrophages can be targeted via CSF-1R inhibitors [45, 46]. PTK2B is a member of the FAK family of tyrosine kinases and is activated by BCR-ABL during CML pathogenesis [47]. Taken together, the inhibition of tyrosine kinases may be a mechanism of YSQHP against MDS.

Yisui Qinghuang powder (YSQHP) is a red ginseng-supplemented derivative of Qinghuang powder (QHP) that has been used to treat MDS for over 30 years [9]. YSQHP induces tumor cell apoptosis and promotes stem cell differentiation to replenish peripheral blood cells, which in turn significantly prolongs patient survival [48]. In addition, arsenic trioxide or realgar triggers the cell cycle arrest and apoptosis of tumor cells by activating the p53 pathway and stimulates hematopoiesis by activating dormant stem cells [49, 50]. The major bioactive compounds of indigo naturalis are indirubin, indigo, and tryptanthrin. Indirubin exerts anti-inflammatory and antiapoptotic effects and used for the treatment of hematopathies [11]. The primary ginsenosides in red ginseng are G-Rg1 and G-Rb1, which show weak antitumor activity. In contrast, the rarer ginsenosides, including G-Rh1, G-Rh2, G-Rg2, and G-Rg3, are more potent [51–53]. For instance, G-Rh2 induces the apoptosis of cancer cells by inhibiting FGFR2, CSF1R, EGFR, and LYN [51]. In addition, G-Rg1 may protect hematopoietic stem cells from oxidative damage by inhibiting the GSK3B/Wnt signaling pathway and can reverse lead acetate- (Pb-) induced toxicity by modulating the senescence-associated p53-p21-Rb signaling network [54]. In a recent clinical trial, MDS patients treated with YSQHP showed improved levels of leukocytes, platelets, and hemoglobin after 4 months of treatment after an initial decrease at 2 months [8].

## 5. Conclusion

CASP8, GSK3B, PRKCA, and VEGFR2 are the potential targets of YSQHP, which potentially inhibits the proliferation of malignant cells and stimulates hematopoietic stem cell differentiation. Furthermore, the antioxidant and antisenescent activities of red ginseng significantly contribute to the antitumor effects of YSQHP. Our findings should be validated experimentally.

## Figures and Tables

**Table 1:** Crude drugs and active components in YSQHP.

Crude drugs	Active ingredient (abbreviation)
Red ginseng (Hongshen)	Ginsenosides Rg1 (G-Rg1)
	Ginsenosides Rf (G-Rf)
	Ginsenosides Re (G-Re)
	Ginsenosides Rd (G-Rd)
	Ginsenosides Rc (G-Rc)
	Ginsenosides Rb1 (G-Rb1)
	Ginsenosides Rb2 (G-Rb2)
	Ginsenosides Rh2 (G-Rh2)
	Di-*n*-octyl phthalate (DNOP)
	Beta-elemene (*β*-elemene)
	Beta-sitosterol (*β*-sitosterol)
Indigo naturalis (Qingdai)	Indirubin
	Indigo
	Isoindigo
	Isovitexin
	Tryptanthrin
	6-(3-Oxoindolin-2-ylidene) indolo[2, 1-b] quinazolin-12-one (qingdainone)
	10h-Indolo, [3, 8-b], quinoline (quinoline)
	Beta-sitosterol (*β*-sitosterol)
Realgar (Xionghuang)	Arsenic trioxide (As_2_O_3_)

**Table 2:** Full names and abbreviations of the 25 target proteins in the DDI-CPI.

Protein name	Abbreviation
Fibroblast growth factor receptor 2	FGFR2
Casein kinase II subunit alpha	CSNK2A2
Lysine-specific histone demethylase 1A	KDM1A
Transitional endoplasmic reticulum ATPase	VCP
Protein kinase C iota type	PRKCI
Tyrosine-protein kinase Lyn	LYN
Vascular endothelial growth factor receptor 2	VEGFR2
Casein kinase II subunit alpha	CSNK2A1
Caspase-8	CASP8
Glycogen synthase kinase-3 beta	GSK3B
Macrophage colony-stimulating factor 1 receptor (CSF1R)	CSF1R
Mitogen-activated protein kinase 9	MAPK9
Protein kinase C beta type	PRKCB
Epidermal growth factor receptor	EGFR
Neutrophil gelatinase-associated lipocalin	NGAL
Protein kinase C alpha type	PRKCA
Protein-tyrosine kinase 2-beta	PTK2B
Cyclin-dependent kinase 2	CDK2
Histone-lysine N-methyltransferase EHMT1	EHMT1
Histone-lysine N-methyltransferase EHMT2	EHMT2
Pirin	PIR
Protein kinase C theta type	PRKCQ
RAC-beta serine/threonine-protein kinase	AKT2
Serine/threonine-protein kinase pim-1	PIM1
Tyrosine-protein kinase HCK	HCK

## Data Availability

The data used to support the findings of this study are available at the following links: traditional Chinese medicine systems pharmacology (TCMSP) (http://tcmspw.com/tcmsp.php); HerDing (http://210.107.182.73/TCMIDWebService/herdingDemo.jsp) database; STITCH database (http://stitch.embl.de/); DrugBank database (https://www.drugbank.ca/); PubChem database (https://pubchem.ncbi.nlm.nih.gov/); GeneCards database (https://www.genecards.org/); Online Mendelian Inheritance in Man (OMIM) database (http://www.omim.org/); DAVID (Database Visualization and Integrated Discovery software (http://david.abcc.ncifcrf.gov)); KEGG (http://www.genome.jp/kegg/) database predicting drug-drug interaction via chemical-protein interactome; or DDI-CPI (https://cpi.bio-x.cn/ddi/).

## References

[1] Ochi Y., Kon A., Sakata T. (2020). Combined cohesin-RUNX1 deficiency synergistically perturbs chromatin looping and causes myelodysplastic syndromes. *Cancer Discovery*.

[2] Chen J., Kao Y.-R., Sun D. (2019). Myelodysplastic syndrome progression to acute myeloid leukemia at the stem cell level. *Nature Medicine*.

[3] Martinez-Hoyer S., Deng Y., Parker J. (2020). Loss of lenalidomide-induced megakaryocytic differentiation leads to therapy resistance in del(5q) myelodysplastic syndrome. *Nature Cell Biology*.

[4] Ramsey H. E., Oganesian A., Gorska A. E. (2020). Oral azacitidine and cedazuridine approximate parenteral azacitidine efficacy in murine model. *Targeted Oncology*.

[5] Mitchell R., Wagner J. E., Hirsch B., DeFor T. E., Zierhut H., MacMillan M. L. (2014). Haematopoietic cell transplantation for acute leukaemia and advanced myelodysplastic syndrome in Fanconi anaemia. *British Journal of Haematology*.

[6] Zhou Q.-b., Yang X.-h., Wang H.-z. (2019). Effect of Qinghuang powder (青黄散) combined with Bupi yishen decoction (补脾益肾方) in treating patients with refractory cytopenia with multilineage dysplasia through regulating DNA methylation. *Chinese Journal of Integrative Medicine*.

[7] Liu B., Song Z., Yu J., Li P., Tang Y., Ge J. (2020). The atherosclerosis-ameliorating effects and molecular mechanisms of BuYangHuanWu decoction. *Biomedicine & Pharmacotherapy*.

[8] Bai W. (2011). *Research on Factors Affecting Curative Effect of Yisui Qinghuang Powder for Treatment of Myelodysplastic Syndromes*.

[9] Hu H. J., Liu F. (2017). Study on the regularity of Yisui Qinghuang powder in the treatment of myelodysplastic syndrome. *Chinese Journal of Woman and Child Health Research*.

[10] Huang Y., Tian Y., Zhang Z., Peng C. (2012). A HILIC-MS/MS method for the simultaneous determination of seven organic acids in rat urine as biomarkers of exposure to realgar. *Journal of Chromatography B*.

[11] Li H., Liu L., Liu C. (2018). Deciphering key pharmacological pathways of Qingdai acting on chronic myeloid leukemia using a network pharmacology-based strategy. *Medical Science Monitor*.

[12] Ru J., Li P., Wang J. (2014). TCMSP: a database of systems pharmacology for drug discovery from herbal medicines. *Journal of Cheminformatics*.

[13] Choi W., Choi C. H., Kim Y. R. (2016). HerDing: herb recommendation system to treat diseases using genes and chemicals. *Database (Oxford)*.

[14] Luo H., Zhang P., Huang H. (2014). DDI-CPI, a server that predicts drug-drug interactions through implementing the chemical-protein interactome. *Nucleic Acids Research*.

[15] Szklarczyk D., Santos A., von Mering C., Jensen L. J., Bork P., Kuhn M. (2016). Stitch 5: augmenting protein-chemical interaction networks with tissue and affinity data. *Nucleic Acids Research*.

[16] Wishart D. S., Knox C., Guo A. C. (2008). DrugBank: a knowledgebase for drugs, drug actions and drug targets. *Nucleic Acids Research*.

[17] Luo H., Zhang P., Cao X. H. (2016). DPDR-CPI, a server that predicts drug positioning and drug repositioning via chemical-protein interactome. *Scientific Reports*.

[18] Reilly B., Tanaka T. N., Diep D. (2019). DNA methylation identifies genetically and prognostically distinct subtypes of myelodysplastic syndromes. *Blood Advances*.

[19] Takahashi K., Patel K., Bueso-Ramos C. (2016). Clinical implications of TP53 mutations in myelodysplastic syndromes treated with hypomethylating agents. *Oncotarget*.

[20] Giles F. J., Stopeck A. T., Silverman L. R. (2003). SU5416, a small molecule tyrosine kinase receptor inhibitor, has biologic activity in patients with refractory acute myeloid leukemia or myelodysplastic syndromes. *Blood*.

[21] Renneville A., Van Galen P., Canver M. C. (2015). EHMT1 and EHMT2 inhibition induces fetal hemoglobin expression. *Blood*.

[22] Magnaghi P., D’Alessio R., Valsasina B. (2013). Covalent and allosteric inhibitors of the ATPase VCP/p97 induce cancer cell death. *Nature Chemical Biology*.

[23] Delfau-Larue M.-H., Klapper W., Berger F. (2015). High-dose cytarabine does not overcome the adverse prognostic value of CDKN2A and TP53 deletions in mantle cell lymphoma. *Blood*.

[24] Huang J., Sengupta R., Espejo A. B. (2007). p53 is regulated by the lysine demethylase LSD1. *Nature*.

[25] Huang H.-S., Liu Z.-M., Hong D.-Y. (2010). Blockage of JNK pathway enhances arsenic trioxide-induced apoptosis in human keratinocytes. *Toxicology and Applied Pharmacology*.

[26] Guo X.-X., Guo Q., Li Y., Lee S., Wei X.-N., Jin Y.-H. (2012). Ginsenoside Rh2 induces human hepatoma cell apoptosisvia bax/bak triggered cytochrome C release and caspase-9/caspase-8 activation. *International Journal of Molecular Sciences*.

[27] Zhang X. W., Yan X. J., Zhou Z. R. (2010). Arsenic trioxide controls the fate of the PML-RAR oncoprotein by directly binding PML. *Science*.

[28] Scaglioni P. P., Yung T. M., Cai L. F. (2006). A CK2-dependent mechanism for degradation of the PML tumor suppressor. *Cell*.

[29] Li P.-F., Li J., Müller E.-C., Otto A., Dietz R., von Harsdorf R. (2002). Phosphorylation by protein kinase CK2. *Molecular Cell*.

[30] Moncada de la Rosa C., Radziwon-Balicka A., El-Sikhry H. (2013). Pharmacologic protein kinase C*α*Inhibition uncouples human platelet-stimulated angiogenesis from collagen-induced aggregation. *Journal of Pharmacology and Experimental Therapeutics*.

[31] Xu H., Czerwinski P., Hortmann M., Sohn H.-Y., Forstermann U., Li H. (2008). Protein kinase C promotes angiogenic activity of human endothelial cells via induction of vascular endothelial growth factor. *Cardiovascular Research*.

[32] Jiffar T., Kurinna S., Suck G. (2004). PKC *α* mediates chemoresistance in acute lymphoblastic leukemia through effects on Bcl2 phosphorylation. *Leukemia*.

[33] Nayak R. C., Hegde S., Althoff M. J. (2019). The signaling axis atypical protein kinase C lambda/iota-Satb2 mediates leukemic transformation of B-cell progenitors. *Nature Communications*.

[34] Baldwin R. M., Garratt-Lalonde M., Parolin D. A. E., Krzyzanowski P. M., Andrade M. A., Lorimer I. A. J. (2006). Protection of glioblastoma cells from cisplatin cytotoxicity via protein kinase C*ι*-mediated attenuation of p38 MAP kinase signaling. *Oncogene*.

[35] Dos Santos C., Demur C., Bardet V., Prade-Houdellier N., Payrastre B., Récher C. (2008). A critical role for Lyn in acute myeloid leukemia. *Blood*.

[36] Slavova-Azmanova N. S., Kucera N., Satiaputra J. (2013). Gain-of-function Lyn induces anemia: appropriate Lyn activity is essential for normal erythropoiesis and Epo receptor signaling. *Blood*.

[37] Yang G., Buhrlage S. J., Tan L. (2016). HCK is a survival determinant transactivated by mutated MYD88, and a direct target of ibrutinib. *Blood*.

[38] Poh A. R., Love C. G., Masson F. (2017). Inhibition of hematopoietic cell kinase activity suppresses myeloid cell-mediated colon cancer progression. *Cancer Cell*.

[39] Bellon M., Lu L., Nicot C. (2016). Constitutive activation of Pim1 kinase is a therapeutic target for adult T-cell leukemia. *Blood*.

[40] Guo Z., Wang A., Zhang W. (2014). PIM inhibitors target CD25-positive AML cells through concomitant suppression of STAT5 activation and degradation of MYC oncogene. *Blood*.

[41] D’Souza S. S., Maufort J., Kumar A. (2016). GSK3*β* inhibition promotes efficient myeloid and lymphoid hematopoiesis from non-human primate-induced pluripotent stem cells. *Stem Cell Reports*.

[42] Ghosh J. C., Siegelin M. D., Vaira V. (2015). Adaptive mitochondrial reprogramming and resistance to PI3K therapy. *Journal of the National Cancer Institute*.

[43] Seetharam M., Fan A. C., Tran M. (2012). Treatment of higher risk myelodysplastic syndrome patients unresponsive to hypomethylating agents with ON 01910.Na. *Leukemia Research*.

[44] Cortes J., Jabbour E., Kantarjian H. (2007). Dynamics of BCR-ABL kinase domain mutations in chronic myeloid leukemia after sequential treatment with multiple tyrosine kinase inhibitors. *Blood*.

[45] Edwards D. K., Watanabe-Smith K., Rofelty A. (2019). CSF1R inhibitors exhibit antitumor activity in acute myeloid leukemia by blocking paracrine signals from support cells. *Blood*.

[46] Bencheikh L., Diop M. K., Riviere J. (2019). Dynamic gene regulation by nuclear colony-stimulating factor 1 receptor in human monocytes and macrophages. *Nature Communications*.

[47] Albano F., Zagaria A., Anelli L. (2013). Gene expression profiling of chronic myeloid leukemia with variant t(9;22) reveals a different signature from cases with classic translocation. *Molecular Cancer*.

[48] Guo X. Q. (2009). *The Clinical Effect of Yisuiqinghuangsan for Treatment of Myelodysplastic Syndrome*.

[49] Zhao S., Zhang J., Zhang X., Dong X., Sun X. (2008). Arsenic trioxide induces different gene expression profiles of genes related to growth and apoptosis in glioma cells dependent on the p53 status. *Molecular Biology Reports*.

[50] Ramirez J.-M., Schaad O., Durual S. (2009). Growth differentiation factor 15 production is necessary for normal erythroid differentiation and is increased in refractory anaemia with ring-sideroblasts. *British Journal of Haematology*.

[51] Qi Z., Li W., Tan J. (2019). Effect of ginsenoside Rh2 on renal apoptosis in cisplatin-induced nephrotoxicity in vivo. *Phytomedicine*.

[52] Cheng Z., Zhang M., Ling C. (2019). Neuroprotective effects of ginsenosides against cerebral ischemia. *Molecules*.

[53] Yan H., Jin H., Fu Y., Yin Z., Yin C. (2019). Production of rare ginsenosides Rg3 and Rh2 by endophytic bacteria from panax ginseng. *Journal of Agricultural and Food Chemistry*.

[54] Li J., Cai D., Yao X. (2016). Protective effect of ginsenoside Rg1 on hematopoietic stem/progenitor cells through attenuating oxidative stress and the wnt/beta-catenin signaling pathway in a mouse model of d-Galactose-induced aging. *International Journal of Molecular Sciences*.

